# The City Doesn’t Sleep: Community Perceptions of Sleep Deficits and Disparities

**DOI:** 10.3390/ijerph16203976

**Published:** 2019-10-18

**Authors:** John Sonnega, Amanda Sonnega, Daniel Kruger

**Affiliations:** 1 Department of Public Health Education, School of Health Promotion and Human Performance, College of Health and Human Services, Eastern Michigan University, Ypsilanti, MI 48197, USA; 2 Survey Research Center, Institute for Social Research, University of Michigan, Ann Arbor, MI 48104, USA; asonnega@umich.edu; 3 Population Studies Center, Institute for Social Research, University of Michigan, Ann Arbor, MI 48104, USA; kruger@umich.edu

**Keywords:** sleep deficits, sleep disparities, community-based research

## Abstract

While sleep research has focused primarily on aspects of the immediate physical environment and behavioral factors, a growing body of evidence suggests that broader social determinants may play an important role in sleep insufficiency. Yet public health education efforts for sleep largely address “sleep hygiene”, with an emphasis on information for getting a good night’s rest. The Flint Sleep Project employed community-based-participatory research methods to try to understand more about the sleep experiences of residents of an urban community reporting sleep insufficiency. The academic and community partner developed recruitment materials with community residents. The focus group protocol also utilized community input. Seven focus groups, with a total of 70 participants, were conducted. When asked about their view of causes for poor sleep, participants identified a range of stressors reflective of social determinants. Economic, safety, and future insecurity were the dominant themes emerging across all seven discussions. Participants also expressed feeling a lack of control over important aspects of their lives. Interventions to improve sleep are more likely to be effective if they include the perspectives of the community. A community-based approach offers opportunities for community empowerment and engagement that can improve efforts at sleep health promotion.

## 1. Introduction

The public health burden of sleep deficits is substantial, with a growing fraction of American adults not getting at least seven hours of sleep on a regular basis [1]. A rapidly growing research literature suggests that sleep deficits—conceptualized here as the personal experience of poor sleep quality and quantity—may be associated with a range of negative mental as well as physical health sequelae, including mortality [1,2,3,4]. Despite its emerging status as a significant health risk factor, sleep has received limited research attention within the field of public health education. The Healthy People 2020 plan [5], which outlines the national health promotion objectives in the United States (U.S.), included sleep health for the first time and will include it in 2030. The broad goal stated in the report is to foster public awareness of the health benefits of adequate sleep. One of the objectives is to increase the proportion of adults who get sufficient sleep, defined as at least seven hours a night. Yet it is not clear what intervention efforts are most effective in communities at risk for sleep problems, specifically geographic areas where epidemiologic research suggests residents may experience worse sleep than other areas. Relatively little research has been done on how people in at-risk communities think about sleep [6,7]. Building our understanding of the social context of poor sleep can provide important information to inform relevant interventions. 

### 1.1. Background

A wide array of social and behavioral factors play a role in sleep outcomes. Many of these are related to individual behaviors and characteristics of the sleep environment (proximal), while others are factors beyond the individual and related more to the conditions in the community (distal). This study utilizes a social determinants theoretical framework to explore community perceptions of sleep in an economically disadvantaged urban area to examine the factors people view as impacting sleep. Individual behaviors, as well as factors in the proximate physical environment, are commonly referred to as part of an individual’s “sleep hygiene”. This includes things like bedroom temperature, cognitive activity, use of medication, external noise, napping patterns, exercise, and practices such as caffeine and alcohol intake [8]. Perceived stress at bedtime is also a risk factor for poor sleep [9,10] to the extent that within the topic of health promotion efforts, sleep hygiene and stress are usually the factors given attention. When stress is addressed in sleep promotion, the focus is on helping the individual “manage” their stress. While such efforts are certainly worthwhile, factors from the more distal social environment are seldom a focus of interventions. 

Social determinants, however, are increasingly implicated in sleep problems. Persistent or recurring adverse events or situations—chronic stressors—are correlated with sleep insufficiency [9,11]. Occupational problems, financial strain, poor relationship quality, and discrimination are associated with poorer sleep outcomes [11,12,13,14,15,16,17]. Broader societal factors, such as longer working hours and technological changes, are thought to contribute to sleep deficits [3,17]. A growing literature investigates the influence of socioeconomic status and race/ethnicity on sleep [13,18,19]. Importantly, residents of urban communities are at increased risk of sleep insufficiency relative to non-urban residents [20].

Epidemiologic research provides some insight into why residents of urban communities might be at higher risk for poor sleep relative to non-urban residents. Sleep insufficiency, for example, has been associated with lower income, higher rates of unemployment, and lower education [21]. Research also suggests that there are racial/ethnic differences in sleep quantity and quality, with African Americans reporting sleeping less well than whites [6,22,23]. Beatty et al. [11] have identified risk factors for poor sleep that may be more common in an urban setting, including environmental factors, such as nearby light and noise, occupational factors such as night shift work, and psychosocial stress, including potential racial discrimination. These social patterns in sleep deficits can be understood as sleep disparities. This is especially important because disparities in sleep might help explain part of the well-documented socioeconomic health gradient [19]. The factors that influence sleep are manifold and interrelated, and the likely mechanisms involved are complex. As noted above, research to date has documented a variety of factors associated with sleep insufficiency [24]. Addressing any single factor in sleep health promotion efforts is unlikely to be effective [25]. Promoting optimal sleep health will require a multifaceted and multilevel approach. Most current sleep health promotion efforts rely on interventions that promote sleep hygiene through educational programs [25,26], but these efforts are limited in their purview. For example, few, if any, focus on the broader socio-structural context as a potential target for intervention. A more complete understanding of the factors that put urban residents at increased risk for sleep problems is needed in order to build effective interventions. 

The community of Flint, Michigan, is an important focus of study as a community that has undergone substantial upheaval and decades of financial decline. Once the home of General Motors (GM), which was a major employer in the community, the city experienced significant economic depression and population loss after GM downsized their workforce in the 1980s. Flint is the largest city in Genesee County, with a population of about 95,000. Approximately 41% of residents live in poverty, and only 12% have a bachelor’s degree or higher. Flint has among the highest crime rates in the country [27,28]. 

### 1.2. A Role for Community-Based Participatory Research (CBPR) in Understanding Sleep

Planning successful public health interventions requires taking a grounded approach that respects people and their context [29,30,31]. Community-based participatory research (CBPR) has emerged as one of the best ways to operationalize this approach in public health [30]. Community CBPR is a collaborative research approach that involves the development of equitable partnerships between academic researchers, community-based organizations, and community members [32]. Community partners are involved in all stages of the research, with the hope of producing outcomes that will benefit the community. The current study, the Flint Sleep Project, utilized CBPR to explore the perceptions of residents of an urban community about reasons for sleep deficits and disparities. The first step in the project was the development of a strong community-academic partnership. Community partnerships are important for developing understanding and designing effective interventions [30,33]. Following the principles of CBPR, the community and the academic partner worked together and with the members of the community on all aspects of the project. The community and academic partner conducted seven focus groups in neighborhoods identified as having sleep deficits and sleep disparities to explore what factors the community viewed as being associated with sleep problems. 

There is little or no published research that addresses the question of what urban residents view as the main factors in sleep deficits and disparities [6]. Making progress on improving the overall sleep health of urban residents will involve gaining further insight into community perceptions about this issue. This approach can offer insights into the complexity of poor sleep and can foster community engagement for efforts to promote better sleep. In this study, we expected that there would be an important connection between sleep and a range of ecological factors, especially feelings of insecurity associated with social determinants [34]. Given the potential importance of sleep in the etiology of specific health problems [3,4,35], addressing the ecological context of sleep may be a promising avenue for not only addressing sleep deficits and disparities but also in preventing the onset of prevalent diseases. 

## 2. Materials and Methods 

### 2.1. Identifying a Sample Area Using GIS Mapping

Data from the Speak to Your Health! Community Survey was used to identify geographic areas reporting sleep difficulties. The survey is designed and implemented by the Prevention Research Center of Michigan (PRC/MI), which is a collaborative partnership involving the University of Michigan, the local health department, health service organizations, foundations, and community-based organizations, as well as individual community members [36]. The survey focuses on health and social issues of concern to residents of Genesee County, Michigan. The authors worked with other members of the PRC/MI to include questions about sleep in the main survey. The questions ask about (1) average hours slept per night, (2) quality of sleep (poor to excellent), and (3) nights per month of trouble sleeping. Geographical Information Systems (GIS) was used to identify zip code areas with sleep disparities based on data from these three sleep variables [37]. 

Three contiguous zip codes in the city of Flint were identified as having comparatively poor sleep relative to the other zip codes in Genesee County, where Flint is located. In the selected area, 51.8% of respondents reported sleeping fewer than seven hours per night, with a mean of 6.48 hours slept per night. Thirty-three percent of respondents in the selected area reported having trouble sleeping at least two weeks out of every month. Finally, 62% reported that they had poor or fair sleep on average in the past month, with the area mean between fair and good. Using GIS mapping, we were able to identify areas with sleep deficits, that is, sleep less than the recommended 7–8 hours per night, and sleep disparities, or sleeping poorly compared to other areas in the county. 

### 2.2. Recruitment and Participants

Focus group participants were recruited with a range of strategies based on the principles of CBPR. The overall recruitment plan utilized the established relationships of the community partner and began with an awareness campaign about the project. The community partner for the present study was a local activist and radio personality with long ties to the Flint community. Flyers with information about the focus groups were distributed at the city’s Neighborhood Round Table and the Community Based Organizations Partnership (CBOP) meeting. The community partner identified locations for focus groups in the selected zip code communities. Arrangements were made to hold focus groups in each area in a variety of settings, including churches, community centers, an apartment complex recreational room, and a rehabilitation center. Recruitment was then conducted individually for each focus group and tailored for that particular neighborhood and location. The community and academic partner met with leaders in each area and location to solicit their help in identifying potential participants and in spreading the word about the focus groups. Flyers were posted with contact information for the project in appropriate locations. The flyers noted that each participant would be provided with a twenty-dollar gift card to a local grocery and/or retail store. 

The goal of recruitment was to assemble a diverse group of participants, with regard to age and gender, from neighborhoods that had reported poor sleep, which the GIS mapping had suggested were predominantly African American majority areas. Participants had to be 18 years of age, but there were no other exclusion criteria. Seven focus groups, ranging in size from 8 to 13 participants, were conducted successively in the spring and summer. A total of 70 individuals took part in the discussions. Six of the focus groups were with community adults, and one was with a group of college students from the area. Most of the sample was African American (93%), and slightly more than half were female (56%). While the respondents were not screened for sleep deficits, they were asked the same two questions used in the survey. Table 1 provides a comparison of the focus group participants to the Speak to Your Health! Survey respondents on these measures. Participants in the focus group slept on average 5.6 hours a night and reported sleep quality, which was the equivalent of fair to good. Focus group participants reported sleeping about an hour less than the survey average for the area, which might indicate self-selection of individuals with sleeping difficulties. All participants reported living in one of the three zip codes identified through GIS mapping.

### 2.3. Semi-Structured Focus Group Protocol

A semi-structured focus group guide was constructed and used for all groups. The content and structure of the guide were developed through discussions with community leaders concerning sleep deficits and disparities. The CBOP helped pre-test the questions and format the guide. This allowed further refinement of the questions for clarity and relevance to the community, as well as improved the transitions and overall flow of the focus group. The global topics covered included: descriptions of sleep, the role of sleep, factors impacting sleep, sleep loss, sleep problems, sleep differences or disparities, and sleep tips. 

Signed informed consent to participate was obtained from each participant at the beginning of each focus group. Time was given to clarify any questions about the study protocol. All participants received a $20 gift card at the completion of the focus group, and beverages and snacks were provided. The Institutional Review Board (IRB) at the University of Michigan at Flint approved the study (HUM00030053).

The average duration of each focus group was approximately one hour. The academic and community partner co-facilitated all focus groups. A student staff member was present as a note taker for all groups. The facilitators reviewed the basic sleep topics to be discussed in the semi-structured guide before beginning the discussion. In all focus groups, all participants were first invited to introduce themselves and to briefly describe their sleep quantity and quality. Participants were encouraged to expand on responses and to exchange opinions with each other. The framework used was exploratory [38] and was aimed at gathering the experiences, opinions, and attitudes of all members of the group.

### 2.4. Analysis

All focus groups were audiotaped and transcribed. The transcripts were checked against the audiotapes by the lead author to ensure fidelity. Using thematic analysis methods [39,40], the content analysis plan for the focus group data followed this structure: familiarization, identifying a thematic framework, indexing, and interpretation. In a thematic analysis, researchers systematically identify patterns or themes that emerge from the data. Following each focus group, the academic and community partner held a debriefing in which they identified emerging themes. Coding categories were derived from the data rather than being selected before analysis. Quotations illustrating relevant opinions or views were flagged as themes emerged. The transcripts were then coded by the lead author and by several research assistants. Discrepancies were resolved by discussion between coders. Two questions generated, by far, the most discussion and accounted for the vast majority of the transcribed material: factors impacting sleep and sleep differences or disparities. Other focus group topics pertained more to specific knowledge of sleep and sleep hygiene. Importantly, knowledge of sleep tips (sleep hygiene) was high overall but did not generate much discussion. Given their salience and prominence overall, the discussions and thematic analysis emerging from the questions around factors impacting sleep and sleep differences or disparities were reported on in the present study. In the context of these questions, aspects of the proximate sleep environment (which relate to traditional sleep tips) did emerge to some extent and were coded as one of the themes in the results.

## 3. Results

The focus groups asked participants, “What are some of the reasons or causes of poor sleep?”. The probe was, “What keeps you awake at night?”. Next, the moderator asked, “Folks in this area of the county tend to report poorer sleep. Do you have any ideas why this might be?”.

The most common first response was “stress”. When prompted, many participants expanded and talked about the relevance of feelings of insecurity in several distinct domains. Following Wilkinson et al. [34], we define insecurity as “the condition of feeling vulnerable or exposed to risk”. Feelings of anxiety, worry, and uncertainty are hallmarks of insecure situations. We considered the term to be an appropriate reflection of several of the themes present in the data. 

Five major themes emerged inductively from the focus group data for these questions. The three most common themes reflect insecurity: economic insecurity, safety insecurity, and future insecurity. Two additional themes indicate the influence of daily settings or routines on sleep: hecticness in daily life and the proximate sleep environment, which is the area immediately surrounding the individual as they try to sleep. Table 2 summarizes the core themes and reports the number of focus groups where the theme was referenced. In this section, we present transcript excerpts that illustrate each core theme in the respondents’ own voices. 

### 3.1. Economic Insecurity

Participants in all of the focus groups spoke of worry related to economics as being one of the main reasons for their difficulty sleeping. Economic insecurity was also suspected by participants as the main cause of sleep disparities in their community. The two major components of economic insecurity were job and financial insecurity, which were seen as clearly linked. The lack of a job (unemployment) or potential loss of a job primarily meant a loss of financial security. Other dimensions of work, such as job demands or work schedule, were mentioned less frequently in relation to sleep. These seemed to be more related to the theme of hecticness and were coded in that category). Similarly, participants spoke about being underemployed, which often meant getting less money than they needed to meet their obligations. Other aspects of economic insecurity mentioned by participants as things that negatively influence sleep were poverty and the local economy. When asked about potential reasons for poor sleep, one respondent provided a brief summary of this theme:

“No money, no car, no job, no sleep.”

The following focus group excerpt represents this theme:

“I think if there were just more jobs in the community, we would sleep better because if you had a nice job, you wouldn’t have to worry about getting a roof over your head, or you know, there’s so many problems that would be alleviated by just having gainful secure employment. Even if you have a job, you worry about losing it. You could go to sleep with peace of mind because you’re employed. So, I think that has a lot to do with sleep differences.”

Many participants recognized the impact of unemployment on sleep disparities in urban settings and the relationship of work to finances and other domains of life.

“There’s lots of contributing factors to people not having good rest, you know. Like, you know, jobs. If you know you’re the head of the household with bills and different things like that. I think if we get more jobs and the financials get better here because of that … I think people will be getting a lot better sleep because they’ll be going to sleep knowing they have a job to wake up to in the morning and they can provide for their families. Until then, I don’t see too much sleep happening.”

Participants also spoke of economic insecurity in a more global sense as being related to sleep deficits and disparities.

“It could just be the economy overall. The worry and stress level is high in the city and you can’t go to sleep.”

Several respondents talked about the current economic climate of the community as something negatively affecting sleep.

“The financial crisis we’re going through makes some areas sleep worse.” “I think when economic change comes, we’ll get more sleep. I think people will get more rest.”

The lack of money itself was also referred to as a reason for poor sleep.

“It all goes back to money.”

“I think we’re trying to get money, trying to survive. You can work two jobs and still be just below the poverty level. You can’t sleep in poverty.”

### 3.2. Safety Insecurity

The second most common theme was anxiety concerning safety for oneself and family members. Respondents indicated how fear of crime, exposure to violence, and neighborhood disorder were associated with problems sleeping. Multiple participants mentioned how hearing “gunshots” or “sirens” often led to difficulties sleeping.

“Gunshots wake you up because you wonder what is going on, so you really can’t go back to sleep.”

“Gunshots, the ambulance, the police. That wakes you up and keeps you up.”

While some respondents mentioned that violence or crime had occurred to them or family members, participant comments were largely focused on apprehension about potential events.

“Worrying about being shot, getting robbed, things like that. That can keep people up and awake. Just fear.”

“The crime-ridden neighborhoods, the gunshots, break-ins, murders. You worry at night.”

“If I leave my car out or something like that, I get up constantly throughout the night, checking to see if my car is still there. We’ve been broken into a couple of times. Even my garage has been broken into, so I get up through the night, check the windows, check the doors, and make sure everything is going good.”

“I have a daughter that got shot. When I know she’s out, I will not sleep until I know she is safely in her house, calls me on the phone. She’s like, ‘Momma, you know I’m twenty-six years old. Stop worrying about me all the time.’ But I do. Are they in the house? Are they safe? I’m not going to sleep until I know she’s in the house.”

In addition to local crime or violence, participants also mentioned neighborhood disorder as relevant to sleep. Neighborhood features, such as dilapidation, presence of gangs, public drug use, and noise, were suggested as reasons for sleep insufficiency in urban settings.

“I’m talking about the drug dealers that be on the corner. They ain’t got no jobs. They’re drinking.”

“Now you drive down the older neighborhoods and whatnot…. It looks like a jungle.”

“The neighborhood looks like a third world country.”

“There are too many problems in the neighborhood. You’ve got gang violence. You’ve got an STD outbreak. It’s too much. So that’s what leads to worse sleep.”

Participants also referenced noise due to groups loitering in the street or neighborhood as being a reason for poor sleep. This category is distinct from more general urban noise such as buses or trains.

“There are kids coming down the street at 1, 2, 3, 4, and 5 in the morning.”

“We have people walking up and down the street all night. You know they haven’t had their rest because they were standing in the street talking all night.”

“The music on the corner is before the shooting, and then the ambulance. So, you are up.”

### 3.3. Future Insecurity

Respondents in the focus groups spoke of concern for the future as something that could negatively affect sleep. This theme intersects with the preceding dimensions but is distinct in reflecting a global worry about the direction of the community. Participants expressed uncertainty about the future and unease with changes in the community. This theme reflects concerns about whether their community can even continue to exist as a community.

“Society has changed over the last twenty or thirty years. Things just aren’t what they used to be.”

“I worry about my kids and societal changes. What will the future be like for them and me? It keeps me awake at night.”

“Just the community in general. There’s not a future for our kids.”

Related to these concerns about the future of their community, participants stated feelings reflecting a loss of control or lack of empowerment within that community. Responses indicated a sense of a loss of personal control in a community that is perceived as being out of control.

“When you have to depend on the city, county, and state to even get you through—you don’t have control of your life. That’s stress. That’s why I can’t sleep.”

“I mean, it just seems like everything is in somebody else’s hands. So, you don’t sleep because you’re worried. You know, there’s just so many things that you don’t feel you’re in control of at all.”

“You are not in control of your life, you know. You don’t know what’s going to happen.”

“You are made to do things that you can’t control your stress level. You know it is hard to control stress.”

“You know everyone is in survival mode and sleep just goes. You are worrying about survival.”

“Like I said, you don’t have no options. What do you worry about? You’re just out of luck basically.”

Future insecurity is interwoven with financial and safety insecurity, and perhaps encompasses the other themes. The following quotation illustrates this relationship:

“I believe that times in the city are so stressed. Everywhere I go, you see it, you know. People are concerned about their jobs, the welfare of their children, their homes. Some people have just lost hope altogether, you see it out there all the time. Just overall lost hope. We can’t sleep because of lost hope.”

### 3.4. Hecticness and Time Pressure

The majority of focus groups referenced how the hectic pace of daily life was associated with sleeping difficulties. Some respondents reported feeling that they had too much going on in their lives.

“We live in what I call the microwave. We want it fast, and we want it now. We be tired enough to get to sleep, but I am going all the time. And I know I got to do this, don’t forget to do that, you know, make sure you do this, make sure you do that. Just going all the time.”

“I just have too much going on. It’s too busy. I can’t sleep.”

“If you’re in a situation that has a lot of activities going on in the environment, you’re not going to be aware that it’s a problem when you don’t sleep. And you’ll start to realize, man, this is the reason why I don’t sleep, because of my environment. If it’s constantly fast-paced, you’re not going to sleep.”

The subject of time pressure was part of the theme of being too busy. Respondents felt that there was not enough time in the day to get tasks accomplished.

“Time. If I have enough time to get things accomplished throughout that day, so I felt that I got this accomplished, then I can go to sleep real well. Time is the issue.”

“It’s not worry. I think that it is time pressure. Too much to do.”

This theme combines elements of having multiple responsibilities and perhaps the lack of resources to carry them out. Respondents also highlighted how a busy environment might lead to rumination.

“Just going all the time. You have so many thoughts in your head. My mind just don’t calm down enough to sleep when I have so much to do.”

### 3.5. Proximate Sleep Environment 

When asked about potential factors associated with sleep difficulties in the community, some participants mentioned issues related to the proximate sleep environment, such as a comfortable bed, noise, and bright lights.

“Mattresses. A bad mattress keep you awake.”

“If you ask people what they want they’ll say mattresses.”

“I slept better in the country because the air was cleaner, didn’t have all the city stink. It just seemed darker; you don’t have all the bright lights.”

“Neighbors got the radio on too loud.”

“I think air pollution and noise pollution is big, too, especially in an area like this.”

This category also focuses on being comfortable enough to be able to sleep. One respondent mentioned the issue of pain. *“I have a pain in my body, so it keeps me from going to sleep. You have to be comfortable”*. As Table 2 shows, this theme was the least frequent to emerge from the data.

## 4. Discussion

This paper reports on an exploratory qualitative analysis of an urban community’s perception of the reasons for sleep deficits and disparities. For participants in the Flint Sleep Project, insecurities emerged as the main factor in sleep difficulties. Focus group participants perceived that worry about economics, safety, and the future was what kept them and others in their community from sleeping. The hectic pace of urban life was also noted. The main themes that emerged are connected by their association with social determinants of health, which are governed by the broader socio-structural and environmental conditions of where people live and their status in society [41]. The multiple negative socioeconomic factors in the Flint community, such as poverty, unemployment, violence, depopulation, unstable housing, substandard education, and pollution, affect a range of health outcomes. 

Offering tips about sleep hygiene is a common public health education approach [25,42], but these factors were much less frequently mentioned. Indeed, the major finding of this study is that when participants talked about reasons for sleep deficits (personal) and sleep disparities (community), economic, safety, and future insecurity were the dominant themes. Sleep may function as an important health marker or indicator for the social determinants of health and may reveal the development of insecurities over community conditions. In the context of a social determinants model, experiencing insecurity reflects conditions in the social and structural surroundings. 

Compared to non-urban, urban settings are at higher risk for sleep problems, for example, shorter sleep duration [43]. Focus group participants perceived this to be largely due to psychosocial stress concerning finances, jobs, crime, and community decline. Research has shown that chronic stressors, such as financial strain [9], occupational problems [13], and neighborhood disorder [44], are associated with poorer sleep. The current study is the first to show that residents in an at-risk community are aware of these associations with sleep insufficiency and perceive them to be of primary importance among the multitude of potential causes. Participants did not appear surprised that urban areas reported poorer sleep and readily discussed the impact of chronic stress and social determinants. Other research has found that individuals in disadvantaged communities are aware of how inequalities affect their health [45]. Our results suggest that the same may be true for urban residents’ perceptions about sleep. For example, quantitative research has shown that poor sleep quality is strongly associated with poverty [46]. Participants in this study echoed that finding. 

Six out of seven focus groups discussed feeling a loss of hope and uncertainty about the future of their community. In this context, many participants talked about feeling like they had no control over events. Hale and Hale [26] have proposed that a lack of autonomy, or control over one’s life projects, is associated with sleep sufficiency. Many characteristics of the urban context are associated with restricted autonomy. This study suggests that people are aware of the association between feeling in control and sleep.

Another theme that arose in most of the focus groups was hecticness or a feeling that there is too much to do and not enough time to do it. Participants thought that feelings of time pressure interfered with their sleep. In modern life, feelings of hecticness are common, but they may be exacerbated in an urban context through feeling less in control and, indeed, in having less control over the means of meeting responsibilities. Here again, the social determinants model can be brought to bear. For example, access to quality transportation and food options are sometimes limited in urban settings where poverty is pervasive. In this circumstance, time pressures may be more acute. It may be harder to “settle a busy mind,” as one respondent suggested, when autonomy is restricted.

Finally, focus group participants talked about matters related to the proximate sleep environment. Some of the factors mentioned, such as having a comfortable bed, would be included in the purview of sleep hygiene. Interestingly, four out of seven focus groups made no mention at all of such concerns. Many resources, such as the Healthy People 2020 evidence-based interventions website [5], refer people to a list of tips about how to get a good night’s sleep. These recommendations include things like: keep a regular sleep schedule, develop a bedtime routine where you relax, keep your bedroom dark, quiet, and comfortable, have a comfortable mattress, pillow and blankets, don’t eat, exercise, have caffeine or alcohol near bedtime, use your bedroom only for sleeping. While these may be useful, they were not the primary reasons stated for sleep deficits and disparities by participants in the Flint Sleep Project. Suggesting that individuals with multiple chronic stressors develop a relaxing bedtime routine to improve their sleep is clearly not enough.

### Implications and Directions for Future Research

The findings of the present study lend strong support to calls for a multi-faceted and expansive approach to promoting optimal sleep health [25,26,29]. Community-based approaches have a rich history in public health in improving adherence and uptake of public health recommendations. Noting that the use of community-based principles is absent in the literature on improving sleep or screening, Johnson et al. [6] state that “Given that planning for Healthy People 2030 is underway, now more than ever, is it crucial to include these objectives from a social determinants of health perspective.” (p. 91). Improving the sleep of at-risk urban communities likely involves understanding the experiences of social determinants. Public health intervention needs to appreciate the views of community members and perspectives on poor sleep outcomes. This study finds that individuals in communities at risk for poor sleep are aware of the social determinants of sleep, identifying insecurities (economic, safety, future) as the primary reason for their experience of poor sleep. A key finding of this study is that individuals are aware of the impact of social determinants on sleep. This is an important starting point for future research.

The next steps could also involve piloting interventions to foster community engagement for promoting better sleep. Such an approach would involve a similar process to that employed in the present study. The key element is the involvement of the community in every step of the process to ensure engagement and, ultimately, an intervention that will be well received and beneficial in the community. The identification of social determinants as a root cause of sleep problems suggests that broad-based public health interventions that strive to reduce the root conditions that produce insecurities, to address social determinants, may prove fruitful in addressing sleep. Mobilizing community partnerships to solve health problems—an essential public health service [41]—would be a step in addressing these conditions. The findings of this study suggest that addressing the economic stability of communities, one of the five key areas of the section on the social determinants of health [5], would be a useful direction and merit future study. 

## 5. Conclusions

CBPR is a practical approach to addressing sleep health in urban communities. This approach aims to foster community engagement and ownership of sleep. In this study, concerns for the social conditions emerged from the CBPR and qualitative research process rather than by being identified by the public health professional. Focus group participants were aware of the broader context of sleep in their community and, during discussions, became engaged in the process of change. Indeed, participants’ expressions of concern about the future of the community demonstrated their commitment to the community; the ultimate goal will be for the community to develop its own solutions. Interventions to improve sleep are more likely to be effective if they include the perspectives of the community. We concur with Hale and Hale [26] that, for people who live in high-risk areas, better sleep is best realized not through sleep hygiene instruction but by improving the conditions in which they live. A community-based approach offers opportunities for community empowerment and engagement that can improve efforts at sleep health promotion. 

## Figures and Tables

**Table 1:** Descriptions of focus group participants compared to the selected area.

Characteristic	Focus Groups	Selected Area
Female	56%	73%
African American	92%	57%
Mean hours of sleep per night	5.6	6.5
Mean sleep quality	3.4	3.1

**Table 2:** Summary of themes.

Theme 1: Economic Insecurity (7 Groups)
Job insecurity Underemployment Unemployment Financial concerns Poverty, lack of resources
Theme 2: Safety Insecurity (6 Groups)
Neighborhood violence Exposure to crime Concern about family safety Neighborhood disorder
Theme 3: Future insecurity (6 Groups)
Community direction Sense of control Loss of hope
Theme 4: Hecticness (6 Groups)
Lack of time Too busy
Theme 5: Sleep environment (3 Groups)
Mattress comfort Room temperature Noise Television
